# Remembering Jan Svoboda: A Personal Reflection

**DOI:** 10.3390/v10040203

**Published:** 2018-04-18

**Authors:** Robin A. Weiss

**Affiliations:** Division of Infection & Immunity, University College London, London WC1E 6BT, UK; r.weiss@ucl.ac.uk; Tel.: +44-208-346-8014

**Keywords:** Rous sarcoma virus, persistent infection, cell transformation, Prague Spring

## Abstract

The Czech scientist Jan Svoboda was a pioneer of Rous sarcoma virus (RSV). In the 1960s, before the discovery of reverse transcriptase, he demonstrated the long-term persistence of the viral genome in non-productive mammalian cells, and he supported the DNA provirus hypothesis of Howard Temin. He showed how the virus can be rescued in the infectious form and elucidated the replication-competent nature of the Prague strain of RSV later used for the identification of the *src* oncogene. His studies straddled molecular oncology and virology, and he remained an active contributor to the field until his death last year. Throughout the 50 years that I was privileged to know Svoboda as my mentor and friend, I admired his depth of scientific inquiry and his steadfast integrity in the face of political oppression.

## 1. Jan Svoboda’s Major Contributions to Tumor Virology

**Figure 1:**
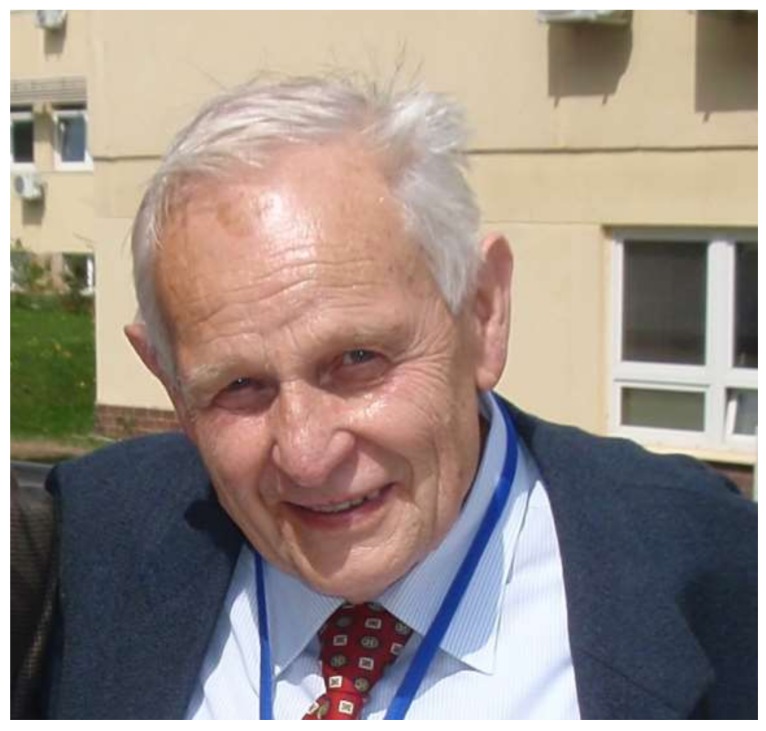
Jan Svoboda at the Centennial Retrovirus Meeting in 2010. Photograph courtesy of Jan Zavada.

Jan Svoboda’s life and achievements have been briefly reviewed in Jiři Hejnar’s excellent obituary [1]. When Svoboda received the accolade of election to the United States (US) National Academy of Sciences as a foreign associate in 2015, he wrote a fascinating perspective on his long career, his scientific discoveries concerning Rous sarcoma virus (RSV), and the political tribulations he faced [2]. I remember him (Figure 1) as one the true pioneers of experimental research on avian retroviruses, alongside Joe Beard, Harry Rubin, Howard Temin, Hidesaburo Hanafusa, and Peter Vogt, whom he met on his first visit to the US in 1963 at a conference on avian tumor viruses organized by Beard at Duke University. Svoboda’s contribution [3] was published in the conference proceedings alongside Temin’s first enunciation of the DNA provirus hypothesis [4].

Jan Svoboda and Howard Temin were born within months of each other in 1934. During the 1960s, their research interest in RSV ran in parallel. Temin used primary chick embryo culture techniques following his and Rubin’s development of the quantitative cell transformation assay as a means of titrating RSV [5]. Svoboda, on the other hand, used in vivo cancer induction by RSV in mammals [6] and the subsequent isolation of immortal cell lines in vitro derived from tumors, the most famous being rat XC cells [7].

Two papers published in 1960 by Temin [8] and Svoboda [6] became early landmarks in tumor virology. They demonstrated that the virus carried a genetic determinant for malignant transformation and viral persistence in tumor cells. It is noteworthy that at the time of publication, RSV was not known to be an RNA tumor virus, because the presence of RNA in the virus particles was not demonstrated until the following year by Lionel and Elizabeth Crawford [9]. Then, Temin drew an analogy between RSV and temperate bacteriophage, postulating not only the conversion of the RNA genome into DNA but also the integration of the DNA genome into host DNA as a “provirus” [4]. The discovery of reverse transcriptase came six years later [10,11].

**Figure 2:**
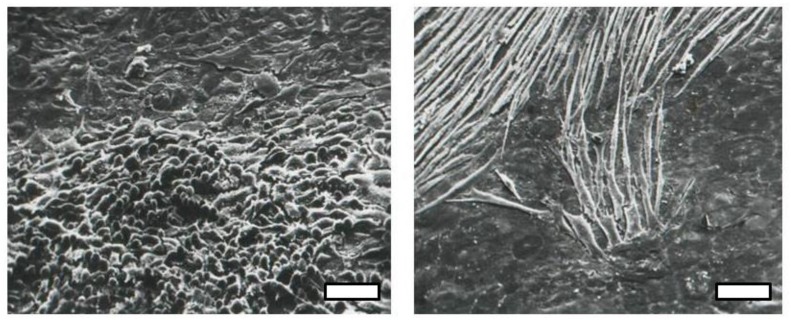
Scanning electron micrographs of round and fusiform foci of Rous sarcoma virus (RSV)-transformed cells [14]. Scale Bar = 30 μm.

Temin’s 1960 paper [8] described how the morphology of RSV-transformed cells is determined by the virus. While the transformed cells in most foci (colonies) have a rounded morphology, occasional colonies of elongated fusiform cells appear (Figure 2). Temin showed that RSV rescued from the fusiform cells “bred true” in generating further fusiform colonies; in other words, the distinctive phenotype was not a result of different cell types being infected in the chick embryo cell monolayer but was a property encoded by the virus. Temin further showed that the transforming activity could be separated from viral replication [12]. These reports represented the first evidence of a transforming gene or oncogene in RSV. It took another 20 years before the biochemical basis of the round and fusiform phenotype was elucidated [13].

Svoboda’s short paper [6] showed that RSV was present in tumors that arose many months after virus inoculation and could be rescued in infectious form by inoculation of X-irradiated tumor cells into chickens. It became apparent that the RSV-transformed rat cells were non-permissive for viral replication yet retained a full, potentially infectious viral genome, a state which Svoboda called “virogenic” [3]. Why mammalian cells infected with RSV are non-permissive for productive infection has not been fully resolved, because the cellular factors that determine RSV activation remain to be identified [2]. Svoboda showed that virus rescue from activation is greatly enhanced by treating mixed cultures of virogenic rat cells and chick embryo cells with inactivated Sendai virus as a fusion agent [15]. Virus production occurs within the heterokaryons formed between XC cells and chick embryo cells [16]. Thus, viral suppression is not governed by a dominant host “restriction factor”, but rather by an avian “permissivity factor” absent from mammalian cells. Svoboda continued to study this question into his later years [2].

Another curious feature of RSV rescued from mammalian cells is that they are replication-competent. The Bryan high-titer (BH) strain of RSV passed exclusively in birds is defective and relies on an avian leukosis virus (ALV) as a “helper” virus to complement a missing *env* gene [17]. However, Svoboda found that the Prague [3] and Schmidt–Ruppin [18] strains replicate without the need of a helper virus, and he surmised that they contain both *env* and *src* as follows:

ALV5′LTR-*gag-pol-env*-3′LTRBH-RSV5′LTR-*gag-pol-src*-3′LTRPrague-RSV5′LTR-*gag-pol-env-src*-3′LTR

All oncogene-bearing retroviruses isolated from birds and mammals are replication-defective except for the RSV strains passed through mammals. The only exception appeared to be the B77 strain of the avian sarcoma virus isolated in Bratislavia [19]. However, later analysis revealed that the structure and phenotype of this virus is identical to Prague-RSV-C, and it therefore seems likely that Prague-RSV became inadvertently substituted for B77 when passaged in the same laboratory. It appears likely that replication-defective RSV preceded the appearance of replication-competent RSV [20], although this could not be tested unless Rous’s original tumors were found.

Svoboda was generous in providing Prague-RSV to all who wished to study it. As reviewed elsewhere [20], the use of replication-competent RSV eventually led to the discovery of retroviral recombination and the identification of the transforming gene later called *src*, its cellular origin [21], and its protein as the first tyrosine kinase to be identified [22]. The precise steps in the transduction of cellular *src* to form viral *src* were investigated [23], but this elegant study did not address the question whether the *env* gene was initially present or absent. It may have been lost in the formation of RSV but regained when high titers of RSV + ALV helper virus were introduced into mammals to induce tumors.

**Figure 3:**
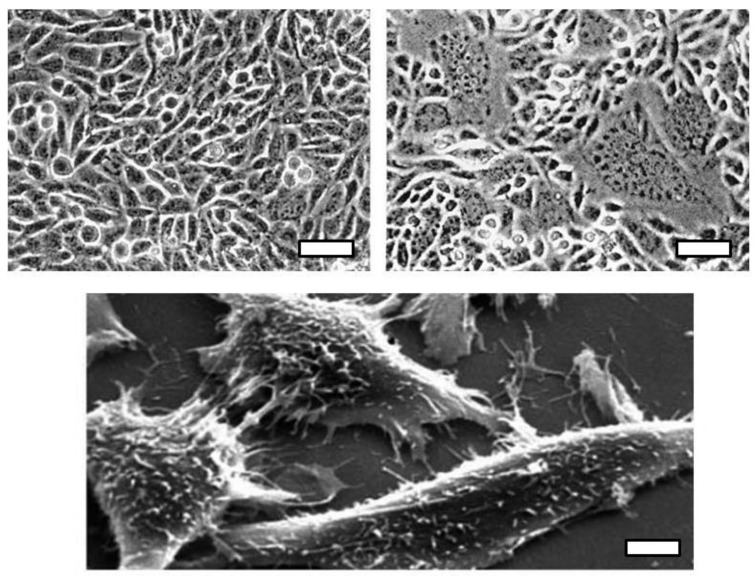
XC rat tumor cells transformed by Rous sarcoma virus. Top, phase contrast micrograph of XC cells in culture (**left**) and after exposure to Moloney murine leukemia virus (**right**), courtesy of Natalie M. Teich. Scale Bar = 25 μm. Bottom, scanning electron micrograph of XC cells, courtesy of Alan Boyde, Robin Weiss, and Pavel Vesely. Scale Bar = 2.5 μm.

Svoboda’s XC cells also proved to be extraordinarily useful to tumor virology. The formal proof of the existence of the DNA provirus came from DNA transfection [24] performed by the Czech émigrés Miroslav Hill and Jana Hillova, who left Prague for Paris soon after the Soviet invasion. Svoboda and Hložanek rapidly confirmed and extended this study [25]. Another protégé was Vaclav Klement who brought XC cells to the NIH in Bethesda where he showed that they could be used in a syncytium assay (Figure 3) for murine leukemia virus [26].

## 2. From the Prague Spring to the Velvet Revolution

Svoboda was born in Prague in 1934, during the period of democracy in Czechoslovakia between the collapse of the Habsburg Empire at the end of World War I and the Nazi invasion in 1938, following the disastrous Munich Agreement. The war years were not too difficult for Svoboda , as he lived mainly at the family’s country home outside of Prague at Dobre Pole (“Good Pasture”), and his parents believed in a liberal education. After World War II, there was a three-year period of coalition government between the communist and democratic socialist parties under Edvard Beneš’s leadership, which Svoboda regarded as a happy state between totalitarian communism and the social callousness of capitalism. However, the fragile democracy was not to last; in February 1948, the Czechoslovak communist party seized total control in a coup d’etat.

In the 1950s, Svoboda studied biology at the Charles University in Prague, and as a student, he volunteered to conduct research on RSV under Milan Hašek’s tutelage at the Czechoslovak Academy of Sciences Institute of Experimental Biology and Genetics (now the Institute of Molecular Genetics), where he was to work for the rest of his career. Hašek was a brilliant if maverick scientist, who discovered the phenomenon of immunological tolerance independently of Peter Medawar in England when he showed the exchange of blood cells by constructing anastomoses between the blood vessels of the chorio-allantoic membranes of pairs of embryonated chick eggs [27]. Vertically transmitted viruses that infect the host before the maturation of the immune system are also not recognized as foreign and hence exist in a state of immune tolerance. Svoboda published his first RSV paper with Hašek on immune tolerance to RSV cells in turkeys [28]. In 1959, he visited Moscow, where he met the pioneering Russian RSV researchers, Lev Zilber, Yuri Svet-Moldavsky, and Fyodor Kisseljov.

During 1967, with the first hints of the relaxation of communist rule, Svoboda gained permission—provided his family remained in Prague—to spend six months with Bob Harris at the Imperial Cancer Research Fund (ICRF) laboratories at Mill Hill on the outskirts of London, for they shared an interest in immunological tolerance to Rous-induced tumors. On 5 January 1968, while Svoboda was in England, Antonin Novotny resigned as President of Czechoslovkia and Chair of the Communist Party to be replaced by Alexander Dubček. At a Party Praesidium in April 1968, Dubček announced the policy to promote “Socialism with a human face”, and thus, the Prague Spring burst into blossom. Alas, this period of liberalization was also short lived when, on the night of 20–21 August 1968, the Soviet leader Leonid Brezhnev sent 200,000 troops and 5000 tanks of the Warsaw Pact to invade Czechoslovakia. Dubček was deposed and was succeeded by the hardliner Gustav Husak. The liberalizing reforms were eliminated step by step, and the country returned to authoritarian rule in almost every aspect of political, social, and economic life.

Hašek was a committed communist, albeit of a liberal disposition, and he influenced Svoboda to join the party in 1966, when Svoboda hoped that scientists and intellectuals might help to reform the regime. Even in his student days, however, Svoboda had distanced himself from Soviet influence on biological theory. He strongly resisted the pressure to pay lip service to the neo-Lamarckian philosophy of Trofim Lysenko that became a strange orthodoxy in the Soviet bloc during Stalin’s later years [27,29]. Lysenko derided both Darwinian evolution by natural selection and Mendelian laws of inheritance. Following the Soviet occupation in 1968, Czech communist party members were required to ratify the presence of the Russian Army in Czechoslovakia. Svoboda flatly and openly refused, and he was promptly expelled from the party. By taking such a stand, he well-understood that he was forfeiting any opportunity for career advancement.

Looking back 50 years, the short-lived Prague Spring was not the only momentous event of 1968 on the world stage. Martin Luther King Jr. and Bobby Kennedy were assassinated, students manned the barricades in Paris and in Berkeley, California, and anti-Vietnam war protests swelled in the US, while apartheid policies intensified in South Africa.

Following the Warsaw Pact invasion, many scientists slipped out of Czechoslovakia to the West, just as Hungarian scientists escaped after the 1956 uprising, and German scientists fled the Third Reich [30]. But, Svoboda remained in Prague and carried on his research as best he could. Although these were difficult times, as he related [2], I nevertheless wonder if a certain degree of isolation and the lack of promotion gave him the space to develop his philosophy and science. Promotion to a position of high responsibility often leaves little time for deep scientific inquiry; it is noteworthy that Gregor Mendel gave up his experiments on inheritance when he was elected abbot of the Augustinian monastery in Brno!

Furthermore, while adversity is hard indeed on the recipient, in posterity, it can bring rewards for the rest of humanity. I have in mind how Alexander Solzhenitsyn’s dire experience of internal exile in the Soviet Union gave the world *One Day in the Life of Ivan Denisovich* and *The Gulag Archipelago*. Similarly, Fyodor Dostoevsky’s incarceration in Tsarist Siberia inspired *From the House of the Dead*, wonderfully turned into an opera by the great Czech composer Leos Janaček. The Belarusian writer, Svetlana Alexievich, remarked in her Nobel Lecture [31] in December 2015: “Suffering is our capital, our natural resource. Not oil or gas-but suffering … Why doesn’t our suffering convert into freedom? Is it truly all in vain?” On the other hand, Pavel Palaček [29] also cites the Talmudic proverb: “We are like olives: only when we are crushed do we yield what is best in us”.

Under the harsh rule of the 1970s, Svoboda indeed managed to yield what was best in him by maintaining links with colleagues in Sweden and the US [32,33]. In the 1980s, when the more oppressive aspects of the regime began to erode slightly, Svoboda was allowed to travel abroad again. He established a fruitful collaboration with Ramareddy Guntaka and spent time as a visiting professor with Guntaka at the University of Missouri [34]. In 1987, when the Soviet leader Mikhail Gorbachev was asked what the difference was between the Prague Spring and his own reforms of glasnost (openness) and perestroika (restructuring), he quipped “Nineteen years”! In 1989, it led to the collapse of communist regimes in Eastern Europe, called the Velvet Revolution in Czechoslovakia, and in 1991, to the end of the Soviet Union and the peaceful separation of Czechia and Slovakia into two independent states.

In the newly liberated Czech Republic, Svoboda was rapidly rehabilitated to assume national leadership in science. He was appointed Director of the Institute of Molecular Genetics and to the council of the Czech Academy of Sciences. He exerted considerable influence on the government to fund research institutes under the Academy’s aegis when their continuation was in doubt. He reformed the Academy’s scientific strategy, and he introduced proper peer review to assess quality of research. These were also quite difficult days, as there was no general consensus on science funding and organization, but Svoboda was held in great respect. He was also immune from gossip and recriminations, being untainted by any hint of collaboration or informing on colleagues during the oppression that had followed the Prague Spring.

Throughout the 1990s, while Svoboda was director, he was able to maintain some laboratory research thanks to loyal support from Jiři Hejnar [35]. After he stepped down as Director of the Institute of Molecular Genetics in 1999 and from his Academy duties, he enjoyed his “retirement” years by readdressing unsolved questions concerning the control of RSV in non-permissive cells [36]. As a younger generation of talented virologists joined Hejnar and Svoboda, they investigated new questions, identifying the cellular receptor of subgroup C avian sarcoma viruses [37] and further analyses of RSV in virogenic cells [38]. These investigations continue, as is evident in this issue of Viruses [39].

Svoboda was instrumental in promoting the Centennial Retrovirus Meeting in Prague in 2010 that celebrated 100 years of the study of Rous sarcoma virus [20]. At that meeting, Svoboda formally pinned to my lapel the Gregor Mendel Medal of the Czech Academy of Science for 2008 and presented the 2010 Medal to Vogt. Since his first paper on RSV in 1955 [28], Svoboda’s research had spanned more than half that era.

## 3. Svoboda’s Influence on My Career

I first met Svoboda in April 1967 at a workshop of the European Tumor Virus Group held in Sorrento, Italy, organized by Giampiero di Mayorca and Claudio Basilico. The biennial workshop alternated between Western Europe and the Eastern Bloc countries and comprised a group of about 60 scientists, including a few Americans. The main focus was on DNA tumor viruses, but RNA tumor viruses were represented (the term “retrovirus” coined by Howard Temin only came after the discovery of reverse transcriptase). The meeting was housed in a beautiful villa looking out across the Bay of Naples; since it poured with rain throughout the two and a half days of discussions, Vesuvius was not visible, and attendance in the meeting room remained full.

This workshop was my first virology conference. During the RNA tumor virus session, Svoboda described his latest analysis of the rescue of infectious RSV from non-permissive, RSV-transformed XC rat cells by cell fusion with chick embryo fibroblasts. Then, I presented my observations on the complementation of envelope-defective Bryan high-titer strain RSV by an endogenous cellular factor, Jim Payne spoke on the Mendelian inheritance in chickens of an antigen cross-reactive with the group-specific antigen (Gag) of ALV, and Peter Bentvelzen spoke on genetic transmission of a determinant for murine mammary tumor virus in the GR strain of mice. These presentations marked the first lines of evidence for endogenous retroviruses inherited through the host germline [40]. Svoboda immediately realized the significance of these findings for Temin’s DNA provirus hypothesis.

It was during Svoboda’s sojourn in London later in 1967 that we got to know each other better. As student at University College London (UCL) in the laboratory of Michael Abercombie (who had discovered contact inhibition of cell division and locomotion and their loss in cancer cells), my main project utilized time-lapse microcinematography to study the behavior of cells transformed by RSV. Svoboda suggested that I should meet his student Pavel Vesely, who also had an interest in microscopy. Vesely came on a week’s visit in November, which initiated a fruitful collaboration [41,42,43]. I made my first visit to Czechoslovakia and spent the 1967/8 New Year with Vesely and the Institute’s Director, Milan Hašek, deep in the snow of the Krkonoše Mountains discussing science and keeping ourselves warm by drinking slivovice!

Vesely brought his RSV-transformed rat cells to London while I pursued chick transformed cells, and we utilized time-lapse microcinematography to study their behavior and proliferation. Alan Boyde, a colleague at UCL who pioneered scanning electronic microscopy (SEM) of hard tissues, such as teeth and bone, wanted to examine cells in vitro using a new technique of coating the cell surfaces with vaporized carbon and gold, so we provided our cultures, resulting in the first images (Figure 2 and Figure 3) of cells by SEM [41].

I then accepted an invitation from Svoboda to spend six months in his group to start on 1 September 1968. My family and I were set to travel to Czechoslovakia when the Soviet tanks rolled into the country on the night of 20 August. What should we do? I tried to contact Svoboda to no avail (there were no emails 50 years ago). Then, on 26 August I received a telegram: “Please postpone visit. Uninvited guests arrived first”.

This clear message was typical of Czech humor in adversity. I did postpone rather than abandon the plan to work with Svoboda and arrived in Prague with my family 11 months later. The Prague Spring was over, and life slowly returned to a dreary “normal” for Czech citizens. There was no accommodation available, but Vesely kindly offered to share his two-room apartment. Between us we had four children under five years old. The bonding forged by this cohabitation seems to have been vertically transmitted, because one of our grandchildren met one of his in Prague last year.

A cancer conference was to be held in Moscow late in August 1969, and an instruction came from the Czechoslovak Academy of Sciences that the Institute must send a fraternal delegate. However, no one volunteered to go to Moscow just one year after the Soviet occupation. Svoboda had a bright idea: might I be willing to represent the Institute? Hašek was delighted, because, in that way, the obligation would be honored, yet national pride would be preserved. I reluctantly agreed and received a visa as the official “Czech” delegate. My train was due to depart on the night of 20 August 1969, exactly one year after the Warsaw Pact invasion. The city was tense with the authorities fearful of demonstrations like those experienced a year earlier in Wenceslaus Square. All buses and trams were halted, and a Soviet tank was stationed on each bridge crossing the Vltava. Svoboda walked me by a circuitous route from the Institute to the main rail station, where he spoke in Russian to the Soviet troops guarding the train. Few others boarded it for the 45-h journey to Moscow.

Svoboda continued to encourage me in many ways. He supported my application for an Eleanor Roosevelt Fellowship to join Vogt’s laboratory in Seattle a year later. At the next European Tumor Virus Workshop held at Smolenice Castle in Slovakia, he introduced me to the virologist Jan Zavada, which led to a lifelong friendship. Zavada subsequently devised pseudotype particles of vesicular stomatitis virus (VSV) bearing retroviral envelopes [44], which proved to be useful for detecting virus receptors and for titrating neutralizing antibodies. Throughout the 1970s, I maintained close contact with both Svoboda and Zavada. With the help of the Director at the ICRF laboratories, Sir Michael Stoker, and the new Director of the Institute of Molecular Genetics in Prague, Josef Řiman, we arranged a formal collaborative agreement between the Czechoslovak Academy of Sciences and the Royal Society. This treaty between academies led to several Czechoslovak scientists coming to London on research visits, including Zavada [45] and Vesely [42] to my laboratory, Vlasta Sovova to John Wyke’s, and Jitka Forstova to Beverly Griffin’s. We arranged to supply reagents not available behind the “Iron Curtain” and in return received valuable antisera and tumor cell lines. Later, we adapted Zavada’s VSV pseudotype technique and devised a syncytial assay akin to the XC test, to identify CD4 as the HIV receptor [46].

It was typical of Svoboda that in 1967, when he was already an established, independent researcher, but I was still a graduate student in Abercombie’s group at UCL, he befriended me as an equal once he perceived our common scientific interest. Looking back, I also realize something that is rare nowadays: although I worked closely with his student Vesely, both in Svoboda’s laboratory in Prague and in London, neither Abercrombie nor Svoboda expected to be co-authors when we eventually published the fruits of our joint endeavor [41,42,43]. Vesely’s and my interests gradually diverged, but in recent years, we have both taken an interest in virological aspects of xenotransplantation [47,48].

The last time my wife Margaret and I met Svoboda in person was at his country home in Dobre Pole, together with Jan and Zuzana Zavada. We discussed science—of course! But with Svoboda’s and Zavada’s breadth of interest and knowledge of Czech culture, we also discussed Secessionist architecture, the art of Alfons Mucha, a concert at the Rudolfinum, and how Svoboda’s favorite book, *The Good Soldier *Švejk**, cheered him up during the harsh years between the Soviet occupation and the Velvet Revolution. We picked Svoboda’s sliva plums and enjoyed plum dumplings prepared by Karin Svobodova and Zuzana Zavadova. I treasure the memory of Svoboda’s immense contributions to Czech and international science. It is also gratifying to see that Svoboda’s vision lives on—including in the philosophical article he co-authored with his son in this issue of *Viruses* [49].
